# *Toxocara* Neuroretinitis Associated with Raw Meat Consumption

**DOI:** 10.4274/tjo.27085

**Published:** 2018-10-31

**Authors:** Irmak Karaca, Jale Menteş, Serhad Nalçacı

**Affiliations:** 1 Ege University Faculty of Medicine, Department of Ophthalmology, İzmir, Turkey

**Keywords:** Toxocara, neuroretinitis, optic neuropathy, raw meat

## Abstract

Neuroretinitis characterized by optic disc edema and star-like exudates in the macula was detected in a patient who presented with sudden unilateral painless vision loss and had a history of raw meat consumption. The patient tested seropositive for *Toxocara*. Combination therapy with steroid and albendazole resulted in an increase in visual acuity and complete resolution of clinical signs.

## Introduction

*Toxocara* infection is a zoonotic disease caused by *Toxocara* canis or *Toxocara* *cati* nematodes. Transmission to humans occurs either through oral intake of eggs in soil contaminated with cat or dog feces, or especially in adults, via consumption of raw or undercooked liver and other meat from animals infected with *Toxocara* larvae.^1^ Ocular toxocariasis is frequently seen in children, although the reported prevalence is also rising in Asian adults in recent years.^2^

Ocular toxocariasis is characterized by chorioretinal granulomas at the posterior pole or at the periphery, focal chorioretinal lesions, and chronic endophthalmitis.^3^ *Toxocara* larvae may also present clinically with vitritis, panuveitis, intermediate or posterior uveitis, and secondary vitreous hemorrhage.^1,4,5,6^ However, optic nerve involvement due to *Toxocara* infection and development of neuroretinitis is rare in the literature.

This report presents the clinical manifestations and treatment results of a patient diagnosed as *Toxocara* neuroretinitis due to consumption of raw meat.

## Case Report

**Figure 1:**
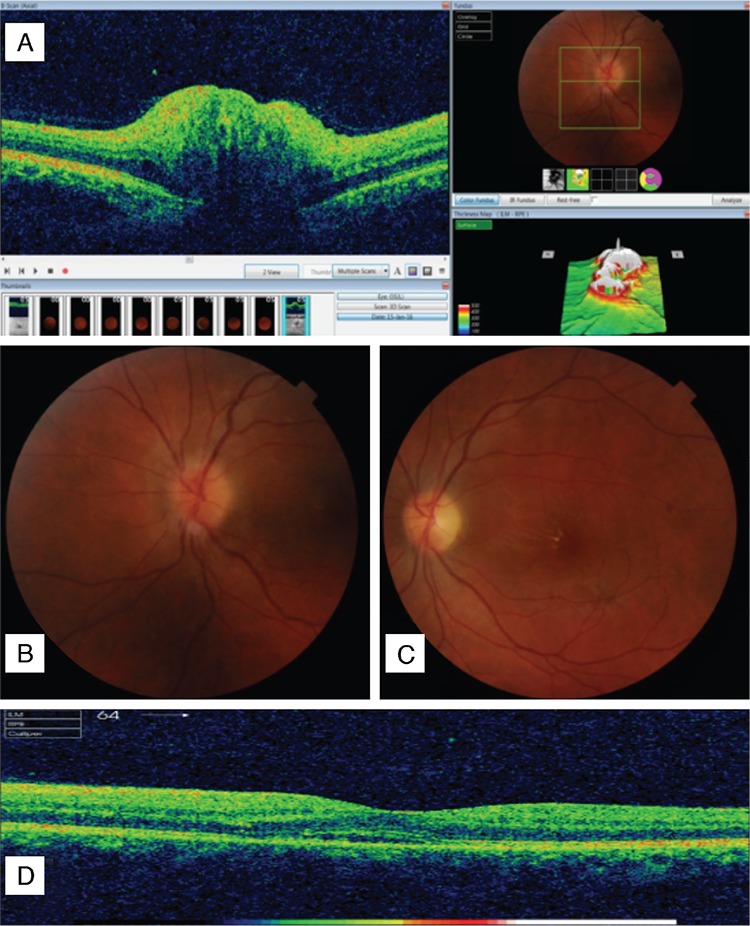
Initial posterior segment findings: A and B) Optic disc edema with indistinct margins; C) Star-like exudates in the macula; D) Subretinal fluid in spectral-domain optical coherence tomography

A 36-year-old male patient presented to our clinic with a complaint of sudden, painless vision loss in his left eye for 1 week. His history was unremarkable except for raw meat consumption. Best corrected visual acuity (BCVA) was 20/20 and 20/125 and intraocular pressure was 16 mmHg and 14 mmHg in his right and left eyes, respectively. Anterior segment examination was normal bilaterally. Pupillary light reflexes showed relative afferent pupillary defect in his left eye. The optic nerve head was edematous with indistinct margins and star-like macular exudates were detected in left fundus examination (Figure 1). In addition, spectral-domain optical coherence tomography (SD-OCT) (Topcon 3D-OCT 2000 Corporation, Tokyo, Japan) showed subretinal fluid in the macula. Right fundus examination was normal. Visual evoked potential was consistent with delayed conduction and Humphrey visual fields showed an inferior arcuate scotoma in the central 20 degrees in the left eye.

Etiological investigation was conducted, including complete blood count, biochemical, viral, bacterial, and parasitological serological tests. Detailed evaluation was performed, including chest x-ray and quantiferon test for tuberculosis, lysozyme and angiotensin converting enzyme level analysis for sarcoidosis, and relevant serological tests for cat-scratch and Lyme disease, along with consultations for rheumatologic and neurological diseases. Cranial magnetic resonance imaging and laboratory tests were all in normal range except *Toxocara* immunoglobulin (Ig) G seropositivity with increased avidity (ELISA and Western Blot) and elevated total IgE (Total IgE = 140 IU/mL) without eosinophilia.

Intravenous methylprednisolone therapy (1 g daily for 1 week) was administered with a preliminary diagnosis of neuroretinitis. After 1 week, BCVA in the left eye increased to 20/30. Considering his history of raw meat consumption, the neuroretinitis was thought to be related to *Toxocara* infection, and oral albendazole treatment (400 mg twice daily) was given in addition to the maintenance corticosteroid regimen for 1 month.

**Figure 2:**
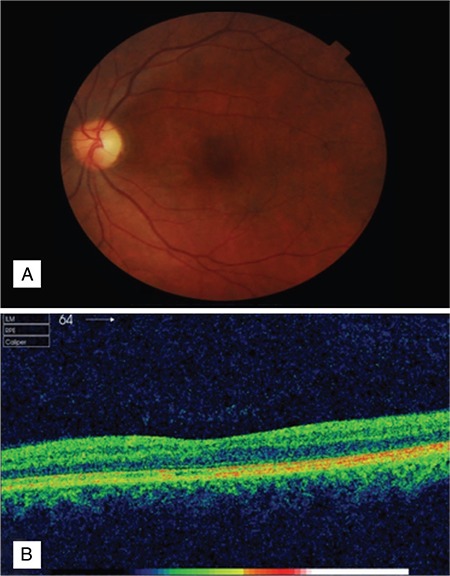
Posterior segment findings after treatment: A) Optic disc and macular region; B) Spectral-domain optical coherence tomography

After 1.5 months, BCVA in the left eye was 20/20 and clinical signs including optic nerve head edema and macular exudates had completely resolved. The subretinal fluid in the macula had also disappeared on SD-OCT (Figure 2).

## Discussion

In this case report, an adult patient with a history of raw meat consumption presented with unilateral neuroretinitis. Detailed investigation revealed that his optic neuropathy was associated with *Toxocara* infection confirmed with Western blot technique. Elevated total IgE levels also supported the diagnosis. Common causes of neuroretinitis such as cat-scratch disease, caused by *Bartonella* species, syphilis, Lyme disease, and toxoplasmosis were all excluded by negative serological test results. In addition, there was no history of exposure to cats. Other possible causes of macular star include hypertensive retinopathy, papilledema, anterior ischemic optic neuropathy, diabetic papillopathy, and toxic etiologies. However, many of these tend to be bilateral, unlike neuroretinitis, and systemic evaluation of our patient was unremarkable for those diseases.

Chronic ocular toxocariasis is usually diagnosed based on clinical findings of granulomas in the retina or at the optic disc together with *Toxocara* seropositivity. Rarely, definitive diagnosis can be made by direct observation of larvae in an ophthalmological examination. For this reason, diagnosis of ocular toxocariasis is presumptive and is usually based on careful history and clinical observation.^3^ Detection of IgG antibodies against *Toxocara* larval antigens with ELISA and confirmation with Western blot technique was also reported to be sufficient for the diagnosis. Chronic increase in total IgE and eosinophilia usually accompany helminthic infections including toxocariasis. However, it was reported that a single larva causing ocular toxocariasis resulted in local eosinophil accumulation in the tissue. Therefore, blood eosinophil count was within normal range in that case.^7^ In our case, total IgE levels were increased and blood eosinophil count was normal.

In the literature, ocular toxocariasis can be seen in adults especially if their history is consistent with raw meat and liver consumption.^2^ Jee et al.^8^ also reported that consumption of raw meat products was more common than history of cat or dog contact in their case series. Thus, history of raw meat consumption along with positive serology also strongly suggested a diagnosis of *Toxocara* neuroretinitis in our patient.

The prevalence of neuroretinitis in ocular toxocariasis was reported as 7.2%.^8^ Yang et al.^9^ related raw meat consumption to *Toxocara* optic neuropathy in their 5 cases, stated as the largest series in the literature. In addition, granuloma in the retina or at the optic disc, peripapillary subretinal exudates, and localized serous retinal detachment along with positive *Toxocara* serology, raw meat consumption, and recurrent vitritis episodes were reported to be distinguishing features of *Toxocara*-related optic neuropathy.

There is no consensus on the management of ocular toxocariasis; however, corticosteroids are usually recommended because they decrease the inflammatory response, thus preventing the development of tractional retinal detachment in these cases.^10^ The use of antihelminthic drugs is controversial, as they may lead to severe reaction in the tissue due to dead larvae. However, albendazole can pass through the blood-retina barrier and reduce recurrence through its intraocular parasiticidal effect. Combination therapy with albendazole and oral prednisolone significantly reduced 6-month recurrence rates as compared to corticosteroid monotherapy and was thought to be successful.^11^ In our case, pulse methylprednisolone therapy resulted in significant improvement in BCVA and the addition of oral albendazole to the regimen resulted in complete resolution of exudates and subretinal fluid.

Ocular toxocariasis has diverse clinical manifestations both in the acute and chronic stages. Thus, clinical signs of neuroretinitis along with a history of raw meat consumption should raise suspicion of *Toxocara* infection.
